# Multi-Laboratory Comparison of Next-Generation to Sanger-Based Sequencing for HIV-1 Drug Resistance Genotyping

**DOI:** 10.3390/v12070694

**Published:** 2020-06-27

**Authors:** Neil T. Parkin, Santiago Avila-Rios, David F. Bibby, Chanson J. Brumme, Susan H. Eshleman, P. Richard Harrigan, Mark Howison, Gillian Hunt, Hezhao Ji, Rami Kantor, Johanna Ledwaba, Emma R. Lee, Margarita Matías-Florentino, Jean L. Mbisa, Marc Noguera-Julian, Roger Paredes, Vanessa Rivera-Amill, Ronald Swanstrom, Daniel J. Zaccaro, Yinfeng Zhang, Shuntai Zhou, Cheryl Jennings

**Affiliations:** 1 Data First Consulting, Inc., Sebastopol, CA 95472, USA; 2 Centro de Investigación en Enfermedades Infecciosas, Instituto Nacional de Enfermedades Respiratorias, Mexico City 14080, Mexico; santiago.avila@cieni.org.mx (S.A.-R.); margarita.matias@cieni.org.mx (M.M.-F.); 3 National Infection Service, Public Health England, London NW9 5EQ, UK; david.bibby@phe.gov.uk (D.F.B.); tamyo.mbisa@phe.gov.uk (J.L.M.); 4 British Columbia Centre for Excellence in HIV/AIDS, Vancouver, BC V6Z 1Y6, Canada; cbrumme@cfenet.ubc.ca; 5 Division of Infectious Diseases, Faculty of Medicine, University of British Columbia, Vancouver, BC V5Z 1M9, Canada; 6 Department of Pathology, Johns Hopkins University School of Medicine, Baltimore, MD 21205, USA; seshlem@jhmi.edu (S.H.E.); yinfengzh@gmail.com (Y.Z.); 7 Division of AIDS, Department of Medicine, University of British Columbia, Vancouver, BC V5Z 1M9, Canada; richard.harrigan@ubc.ca; 8 Research Improving People’s Lives, Providence, RI 02909, USA; mhowison@ripl.org; 9 National Institute for Communicable Diseases, Johannesburg 2192, South Africa; gillianh@nicd.ac.za (G.H.); johannal@nicd.ac.za (J.L.); 10 National HIV and Retrovirology Laboratories at JC Wilt Infectious Diseases Research Center, Public Health Agency of Canada, Winnipeg, Manitoba R3E 3R2, Canada; hezhao.ji@canada.ca (H.J.); emmar.lee@canada.ca (E.R.L.); 11 Division of Infectious Diseases, Brown University Alpert Medical School, Providence, RI 02912, USA; rkantor@brown.edu; 12 IrsiCaixa AIDS Research Institute, Badalona, 08916 Catalonia, Spain; mnoguera@irsicaixa.es (M.N.-J.); rparedes@irsicaixa.es (R.P.); 13 Center for Research Resources-Immunology Reference Laboratory, Ponce Health Sciences University-Ponce Research Institute, Ponce, PR 00716, USA; vrivera@psm.edu; 14 Lineberger Comprehensive Cancer Center, University of North Carolina, Chapel Hill, NC 27514, USA; ron_swanstrom@med.unc.edu (R.S.); shuntaiz@email.unc.edu (S.Z.); 15 RTI International, Research Triangle Park, NC 27709, USA; dzaccaro@rti.org; 16 Rush Medical College, Chicago, IL 60612, USA; cheryl_jennings@rush.edu

**Keywords:** HIV-1, drug resistance, genotyping, NGS

## Abstract

Next-generation sequencing (NGS) is increasingly used for HIV-1 drug resistance genotyping. NGS methods have the potential for a more sensitive detection of low-abundance variants (LAV) compared to standard Sanger sequencing (SS) methods. A standardized threshold for reporting LAV that generates data comparable to those derived from SS is needed to allow for the comparability of data from laboratories using NGS and SS. Ten HIV-1 specimens were tested in ten laboratories using Illumina MiSeq-based methods. The consensus sequences for each specimen using LAV thresholds of 5%, 10%, 15%, and 20% were compared to each other and to the consensus of the SS sequences (protease 4–99; reverse transcriptase 38–247). The concordance among laboratories’ sequences at different thresholds was evaluated by pairwise sequence comparisons. NGS sequences generated using the 20% threshold were the most similar to the SS consensus (average 99.6% identity, range 96.1–100%), compared to 15% (99.4%, 88.5–100%), 10% (99.2%, 87.4–100%), or 5% (98.5%, 86.4–100%). The average sequence identity between laboratories using thresholds of 20%, 15%, 10%, and 5% was 99.1%, 98.7%, 98.3%, and 97.3%, respectively. Using the 20% threshold, we observed an excellent agreement between NGS and SS, but significant differences at lower thresholds. Understanding how variation in NGS methods influences sequence quality is essential for NGS-based HIV-1 drug resistance genotyping.

## 1. Introduction

Next-generation sequencing (NGS) is increasingly used in molecular diagnostic laboratories, including for HIV-1 drug resistance (HIVDR) genotyping [1,2,3,4]. NGS methods have several potential advantages over standard Sanger sequencing (SS) methods, including more a sensitive detection of low-abundance variants (LAV, here defined as variants detectable by NGS but not SS), potentially less subjective and more quantitative and automatable data processing steps, and a reduction in cost. Since virus populations within individuals include multiple variants (possibly including variants with mutations conferring drug resistance) that are present at frequencies below the minimum required for detection by SS, NGS has the potential to improve the utility of HIVDR genotyping [5,6]. However, NGS involves complex laboratory and analytic methods that are not yet well-standardized between laboratories, although recommendations for bioinformatic analysis pipelines have been proposed [7,8,9]. HIVDR genotyping tests based on different NGS platforms are also commercially available [10,11,12,13]. The clinical significance of LAV is largely unknown, although there is a general agreement that LAV detected by more sensitive methods such as NGS may increase the predictive value of HIVDR genotyping for clinical outcomes as compared to SS [14,15,16,17]. There is ongoing debate, along with a general lack of certainty, regarding the optimal LAV threshold for clinical applications [17,18,19].

The World Health Organization (WHO) HIVDR Laboratory Network supports the national surveillance of HIVDR in low- and middle-income countries (LMIC) [20,21,22]. Network laboratories currently employ a variety of SS-based methods including commercial kits and in-house developed procedures, but several laboratories are planning to adopt NGS methods. Since resistance prevalence trends over time and between countries and geographic regions are an important part of the survey results, the standardization of genotyping assay performance characteristics is crucial. Consistency is ensured by the implementation of a rigorous validation, quality assurance, and quality control system [23,24,25]. New technologies, such as NGS, must be introduced carefully, with consideration given to comparability to results from other laboratories in the network and to historical data. Currently, most laboratories located in LMIC do not have access to NGS platforms. Until all WHO Network laboratories have the capability to implement NGS, individual laboratories that are doing so are required to report consensus sequences that mimic those generated by SS as closely as possible. To support this approach during this transitional period, a standardized threshold for reporting LAV that generates data comparable to those derived from SS is needed.

The National Institute of Allergy and Infectious Diseases (NIAID) Virology Quality Assurance (VQA) program provides a comprehensive quality assessment program for virologic assays for HIV, including drug resistance genotyping [26]. A crucial function of the VQA program is to ensure the validity and inter- and intra-laboratory comparability of virologic laboratory data generated for NIAID-supported clinical trials and research by the provision and analysis of proficiency testing panels. The VQA program also implements standards of performance for existing and state-of-the-art new virologic assays, develops and evaluates biostatistical methods relating to the assays, and acquires, tests, stores, and dispenses quality control materials and reagents. Since 2007, the VQA has provided proficiency testing specimens to the WHO HIVDR Laboratory Network [24]. This resource is well-suited to the investigation of NGS LAV thresholds that maximize the comparability of sequences from SS assays.

In this paper, we report for the first time an inter-laboratory comparison of HIV protease and reverse transcriptase sequences from an external quality assurance panel, comparing NGS sequences to Sanger sequences and NGS sequences between laboratories.

## 2. Materials and Methods

### 2.1. Specimens

Ten VQA HIVDR genotyping proficiency testing panel specimens (five from each of the two panels) were used. The specimens were prepared from patient plasma or cell culture virus stocks, and belonged to HIV-1 subtypes B, C, D, or F, at viral load loads ranging from 3656 to 29,139 copies/mL. Several specimens contained multiple drug resistance-associated mutations (DRMs), some of which were present as mixtures (Table 1).

### 2.2. Sequencing Methods

Ten laboratories participated in this evaluation study. The laboratories are numbered from 1 to 10. Six of the laboratories were from the WHO HIVDR Laboratory Network, and four were extra-network laboratories with extensive HIVDR testing and NGS experience. Each laboratory used its own RNA extraction, RT-PCR amplification, raw sequencing data analysis, and post-testing QA procedures (Table A1), but all used the Illumina MiSeq platform (Foster City, CA, USA). One laboratory (#1) used a unique molecular identifier approach to more accurately quantitate the number of amplified templates in each reaction [27,28].

The laboratories submitted consensus sequences for each specimen using LAV thresholds of 5%, 10%, 15%, and 20% (i.e., minor nucleic acid variations with frequencies below these thresholds were ignored, and all variations with frequencies above the threshold are included in the base call at that position). Lower thresholds were not evaluated because of the lack of data demonstrating the clinical relevance of LAV at less than 5%. The software used to generate the consensus sequences was not able to do so using the 20% threshold in laboratory 4, so there are no data for this laboratory in the 20% group. The consensus sequences spanned the protease (PR)-reverse transcriptase (RT) regions that encompass all DRM sites that contribute to the resistance to PR and RT inhibitors of interest to the WHO HIVDR surveillance program (PR 10–93 and RT 41–238), except those from laboratory 1 which did not cover RT amino acids 123 to 151.

### 2.3. Sequence Comparison

The SS consensus sequences for each specimen were generated by VQA based on over 30 results from independent laboratories that used an SS-based, FDA-approved commercial genotyping kit (ViroSeq or TruGene), using an 80% identity threshold. Where an 80% absolute agreement was not reached, an “N” was inserted at that position, and these positions were excluded from identity percentage calculations. The VQA SS consensus sequence covers protease codons 4–99 and reverse transcriptase 38–247; portions of the NGS sequences outside this region were excluded from the analysis of identity to the VQA consensus. A secondary analysis evaluating only the sequence at DRM codons (any position with a potential impact on the penalty score in the Stanford HIVdb algorithm, version 8.5) was also performed.

The sequences were aligned using Geneious software (version 11.1; San Diego, CA, USA) and analyzed in Microsoft Excel. To assess the extent to which the sequences between labs agreed with each other, without comparison to SS, the sequence identity at all positions was determined between all possible pairs of sequences for each specimen and threshold. Missing data (gaps) were ignored.

Sequence quality evaluation (i.e., assessing the presence of anomalies such as frameshifts, stop codons, APOBEC mutations, and unusual mutations) was performed with Stanford HIVdb (https://hivdb.stanford.edu/). The anomalies reported in the region not covered by laboratory 1 (RT 123–151) were ignored for this laboratory only.

Comparisons of percent identity between thresholds were performed using the Wilcoxon matched-pairs signed rank test and paired t-test (Prism 7, GraphPad, San Diego, CA, USA), and a random effects model with laboratory and specimen as random effects, cut-off values as fixed effects, and pairwise adjusted estimates of differences between cut-off values using SAS PROC MIXED.

## 3. Results

### 3.1. Comparison of NGS Sequences to VQA Sanger Consensus

Six of the ten laboratories generated results from all ten specimens. Three laboratories (4, 7, and 9) did not report results for one specimen each, and laboratory 8 did not report data for three specimens. There was no obvious association between assay failure and virus subtype or viral load.

**Figure 1:**
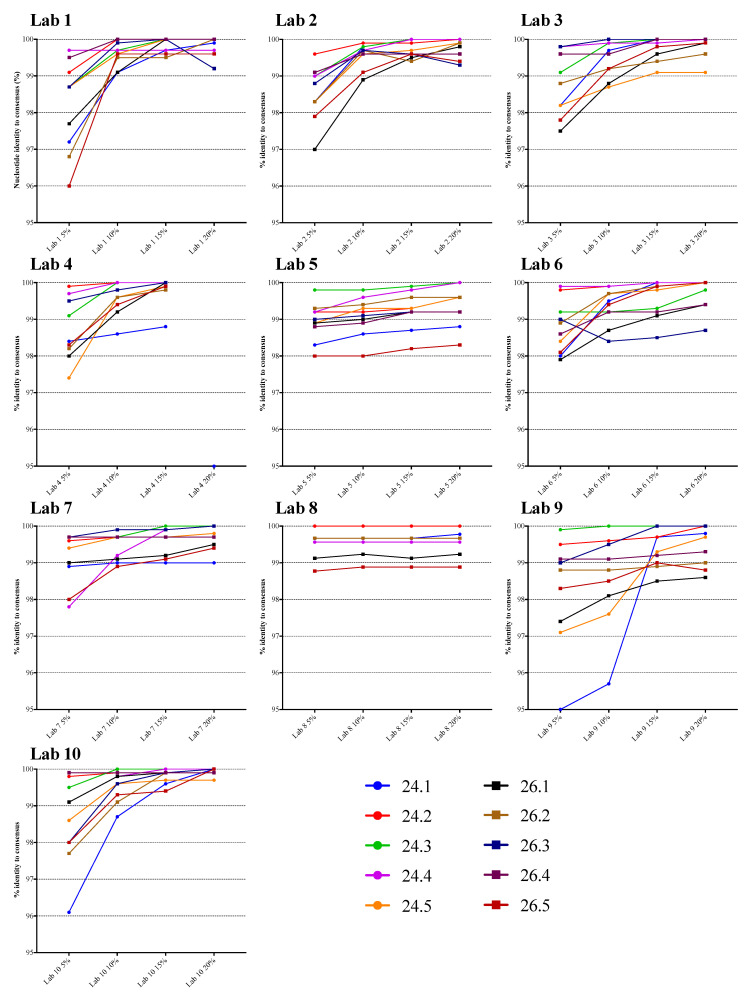
Plots of next-generation sequencing (NGS)-derived PR-RT nucleotide sequence identity vs. VQA Sanger consensus at various thresholds. Each line represents one specimen from panel 24 (24.1 through 24.5) or 26 (26.1 through 26.5).

The NGS sequences generated using the four different thresholds were compared to the VQA SS consensus sequence. Figure 1 displays the percentage nucleotide identity for each specimen, and for each laboratory. There was a strong tendency for the percent identity to increase as the threshold increased, especially in laboratories 1, 2, 3, 4, 9, and 10. The identity appeared to increase substantially from 5% to 15%, then increase only slightly at 20%. In laboratories 5, 6, 7, and 8, there was relatively less impact of threshold on agreement with the SS consensus for most specimens, and the identity at the lower thresholds was higher than in the other laboratories. For example, the mean percent identity at the 5% threshold was 99.5% in laboratory 8 and 99.1% in laboratory 7, compared to 98.2% in laboratories 1 and 9. The overall percent identity was slightly lower for specimen 24.1, which had 2.3% mixtures and a viral load of 7815 copies/mL.

The mean percent nucleotide identity with the VQA SS consensus sequence across all results (specimens and laboratories) at the four different thresholds was highest at the 20% threshold (99.7%, the range in values was 98.3–100%), compared to 15% (99.6%, range of 98.2–100%), 10% (99.4%, range of 95.7–100%) or 5% (98.7%, range of 95.0–100%) (Table A2). In general, and as expected, the increased number of mismatches in the NGS sequences was a result of more mixed bases in the lower threshold NGS sequence but not in the VQA SS consensus. These mixed bases at lower thresholds may indicate LAV that SS is unable to detect, or they may reflect sequencing or analytic errors. All comparisons of percent identity between thresholds were statistically significant (*p* < 0.0001, Wilcoxon test or paired *t*-test; *p* < 0.01, random effects model; Table A3). The mean identity of each laboratory’s sequences was over 99% for all laboratories at the 15% and 20% thresholds, nine of ten at 10%, and two of ten at 5%.

The analysis of nucleotide sequence identity compared to SS was also performed considering only HIVDR-associated codons. The observed trends were similar, although the differences between the 15% and 20% thresholds were smaller.

### 3.2. Comparison of NGS Sequences Between Laboratories

**Figure 2:**
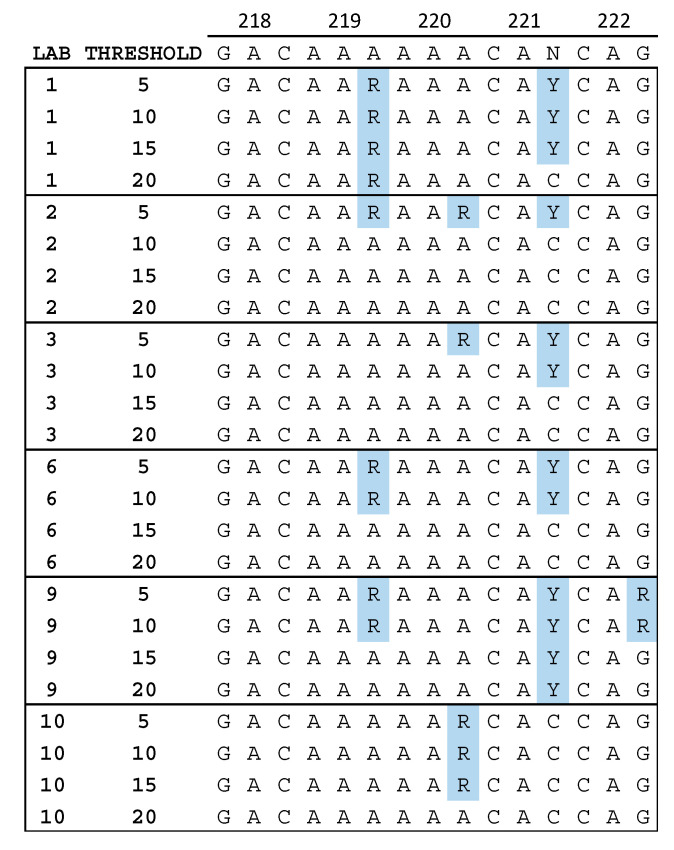
Nucleotide sequence alignment for six laboratories. The VQA Sanger sequencing (SS) consensus is shown at the top. Mixtures of A and G (R) or C and T (Y) that were reported by some but not all laboratories are highlighted in blue. The sequences from laboratories 5, 7, and 8 did not contain any mixtures in this region, and those from laboratory 4 contained the Y in codon 221 at all thresholds reported (5%, 10%, and 15%).

While analyzing the level of identity between NGS and SS consensus sequences, we observed that the positions at which differences were located were not always the same between laboratories. Figure 2 shows a portion of the sequence alignment for specimen 24.1 in reverse transcriptase between amino acids 218 and 222. Mixed bases in the third position of codon 219 and 220 were more often detected at lower thresholds, but in different codons for different laboratories. This suggests that the reduced agreement at low thresholds described above is not simply a result of a consistent increase in sensitivity for the detection of LAV by NGS, but that variation in LAV detection between laboratories may be position dependent—especially at low thresholds.

**Figure 3:**
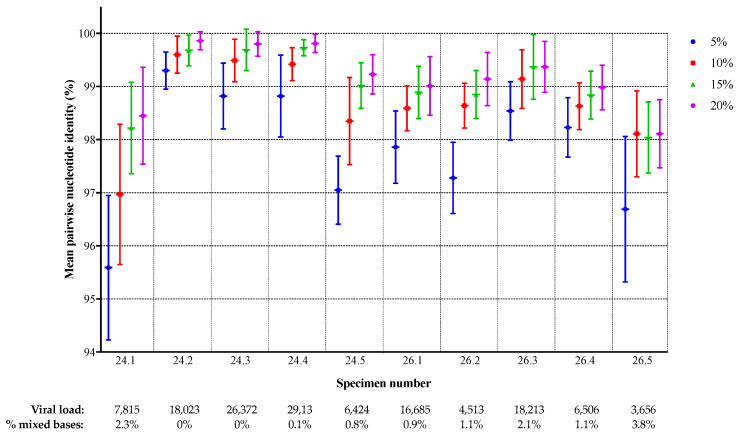
Protease/reverse transcriptase nucleotide sequence concordance between laboratories. The mean percent identity with standard deviation is shown for each specimen and threshold.

To assess the degree of inter-laboratory sequence agreement, the individual sequences for each specimen were compared to the corresponding sequences from the other laboratories, for each specimen and threshold, and the pairwise sequence identity was calculated. The number of comparisons ranged from 28 (because of missing data from some laboratories) to 45. The mean percent identity between laboratories for each specimen and threshold is shown in Figure 3. There was a clear increase in sequence agreement as the threshold was raised for all specimens, except 26.5 for which the results for 10%, 15%, and 20% were similar to each other, but still higher than for 5%. The highest inter-laboratory agreement was observed for specimens 24.2 (99.9% at the 20% threshold), 24.3, and 24.4 (both 99.8%) while less agreement was observed for specimens 24.1 (98.4%) and 26.5 (98.1%). There was some correlation between the level of agreement and sequence heterogeneity in the specimen: specimens 24.2, 24.3, and 24.4 had no or very few mixed bases in the VQA SS consensus sequence, while 24.1, 26.3, and 26.5 had mixtures at more than 2% of positions (Table 1 and Figure 3). Specimen 26.5 had the lowest concordance overall, as well as the lowest viral load and the highest proportion of mixtures in the VQA SS consensus.

### 3.3. Quality Assurance Anomalies

**Figure 4:**
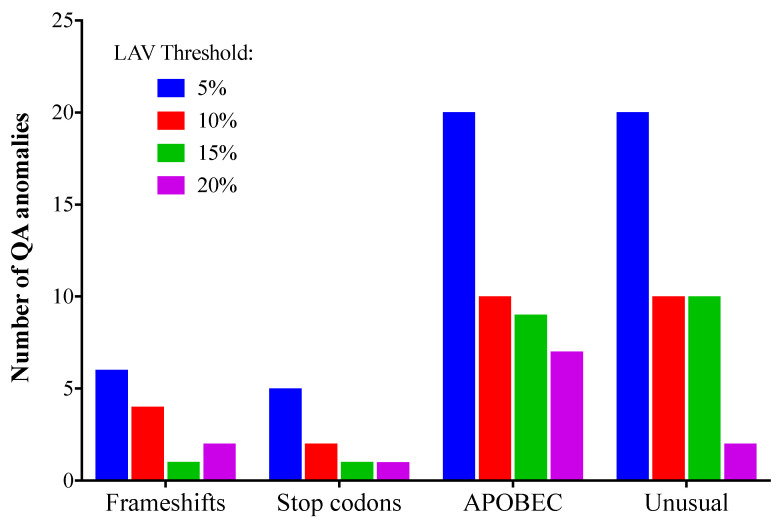
Sequence quality assurance anomalies (total for all laboratories) at different low-abundance variant (LAV) thresholds. Sequence quality evaluation was performed with Stanford HIVdb (https://hivdb.stanford.edu/). HIVdb sequence analysis was performed using NGS sequences generated using the 5%, 10%, 15%, or 20% threshold levels.

At low NGS mutation detection thresholds, it is more likely that sequence anomalies resulting from RT-PCR error and/or host DNA editing enzymes will be detected [29]. Sequence anomalies include frameshifts, stop codons, APOBEC mutations, and “unusual” mutations (amino acid changes that have only rarely or never been observed before in the Stanford HIV sequence database). This could be at least partly a result of artefactual codon sequences that can occur when two or more bases in the codon are mixed; for example, “YGR” may in fact be a mixture of TGG (tryptophan) and CGA (arginine), but could also be translated as TGA (stop) or CGG (arginine). The total number of sequence anomalies for all laboratories detected at 5, 10, 15, or 20% using the Stanford HIVdb sequence analysis tool was 51, 26, 15, and 12, respectively. The numbers of each type of anomaly are shown in Figure 4. All four types of QA anomalies were most common when the 5% threshold was used, and least common at 15% or 20%. No QA anomalies were found in the VQA SS consensus sequences, apart from those that resulted from the “N” where 80% consensus was not reached.

## 4. Discussion

The global surveillance of HIVDR relies on high quality, standardized methods for detecting DRMs in specimens from survey participants. The current standard platform method is SS, and until NGS is accessible to all laboratories that are contributing sequence data for HIVDR surveys, those that adopt NGS-based methods must be able to produce sequences that have the same performance characteristics as laboratories using SS. It is recognized that this transitional approach may initially lead to the under-utilization of some potential advantages of NGS, including better sensitivity for LAV detection and de-convolution of complex mixtures.

We evaluated several thresholds for reporting LAV from NGS data and demonstrated that the similarity to SS data was highest when a 20% threshold was applied. Furthermore, inter-laboratory comparability was also highest at this threshold. Previous studies that evaluated the sensitivity of SS for LAV detection or that compared sensitive point mutation assays and SS are consistent with the 20% threshold [30,31,32,33].

The decreased agreement between NGS and SS data at thresholds below 20% might have been considered predictable, based on the concept that additional mixtures are expected to be reported in the NGS sequences, as LAV present at low frequency are detected more frequently. However, we found that the observed decreased agreement below 20% is not solely the result of the better sensitivity of NGS, since the inter-laboratory agreement also decreased as the threshold was lowered. These observations suggest that the detection of LAV can be subject to stochastic effects that may not be robustly repeatable or reproducible between methods or laboratories. Importantly, our results raise concerns about accuracy and inter-laboratory (and perhaps also intra-laboratory) reproducibility at low thresholds such as 5%, and strongly suggest that if even lower thresholds were to be used, the reproducibility would continue to decline. In the future, if the clinical significance of drug resistant LAV is conclusively shown to increase the predictive value of HIVDR genotyping for clinical outcomes and a threshold below 20% is established, there may be enough impetus to transition laboratory assays that support the public health surveillance of HIVDR to NGS platforms. At that time, it will be important to gain a better understanding of the sources of inter-laboratory variability in sequence determination and implement ways to minimize their impact. Both processes would be greatly facilitated by the development and use of standardized reference and/or control material with relevant LAV at specific frequencies, for use in external QA programs and/or assay optimization and validation. Other challenges inherent in the capacity of laboratories in LMIC to perform NGS-based HIVDR genotyping (e.g., instrument cost, operator training, and the availability of technical support) will also require attention and significant resources.

The low inter-laboratory reproducibility of NGS sequences may also be at least partly related to input amplifiable copy number, specimen sequence heterogeneity, position in the genome (Figure 2 and Figure 3), and differences in the bioinformatics pipelines used. This complexity strongly suggests that clinical specimens with these characteristics should be included in external QA programs and inter-laboratory comparisons, rather than virus clones or reconstructed mixtures. With regard to differences in pipelines, Lee et al. [34] evaluated the data from six of the ten laboratories included in this study using five pipelines and reported that sensitivity was good (over 99%) using thresholds as low as 1%, but specificity was low (82.4%) at the 1% threshold; they therefore suggested that a 2% threshold would be more reliable than 1%.

Our study has several limitations. (1) One or more of the NGS methods used may include unique aspects that make them more accurate than others or than those used to generate the SS standard comparator sequences (for example, the use of unique molecular identifiers, different input RNA volumes, or bioinformatic analysis pipelines). In this case, that method might generate sequences that are very different from the gold standard, but in reality, closer to the correct result. (2) Because thresholds over 20% were not evaluated, it is possible that the optimal threshold is higher; it is expected that at very high thresholds, a decrease in concordance would be seen, as mixtures start to be *under-*called. (3) We have analyzed similarity to SS across the entire sequence uniformly; different optimal thresholds may exist for specific DRM positions, due to the context dependence of chromatogram peak height in SS raw data. For example, a LAV that involves a change from a “weak” A base to a “strong” G might be expected to reach maximum identity at thresholds lower than 20%. (4) It is possible that many of the sites where the variability between laboratories is introduced involve synonymous mutations that would not have any impact on the predicted amino acid sequence and DR interpretation. (5) All participating laboratories used the Illumina MiSeq platform, limiting the application of our conclusions to that platform. Finally, (6) several assay variables that could be hypothesized to have an impact on NGS assay reproducibility have not been explored, including PCR reaction input copy number, sampling bias related to procedural bottlenecks, PCR-associated errors, and analysis pipeline methodology.

## 5. Conclusions

Of the LAV thresholds tested here, 20% led to PR-RT NGS consensus sequences that matched SS most closely and had the highest level of inter-laboratory agreement. Using the 20% threshold, we observed excellent agreement between NGS and SS, but significant differences at lower thresholds that may limit their use for global surveillance of HIVDR. Understanding how variation in NGS methods influences sequence quality is essential for NGS-based HIV-1 drug resistance genotyping and other applications where LAV detection reproducibility is important.

## Figures and Tables

**Table 1:** Characteristics of Virology Quality Assurance (VQA) specimens used in this study.

Specimen	Viral Load ^a^	Subtype ^b^	PR DRMs ^c^	RT DRMs ^c^	% Mixed Bases in SS Consensus ^d^	Number of Amplification Failures
24.1	7815	B	None	T215C	2.3%	0
24.2	18,023	F	K20R, M36I	None	0.0%	0
24.3	26,372	C	M36I	M41L, V75T, V90I, V106M, V179D	0.0%	0
24.4	29,139	C	M36I	M41L, K103N, M184V, T215Y	0.1%	1
24.5	6424	B	L10I, L33F, M46L, I54V, A71I/T, V82A, L90M	M41L, E44D, A62V, D67N, L74V, L100I, K103N, H208Y, L210W, T215Y H221Y	0.8%	1
26.1	16,685	C	M36I, T74S	D67N, K70R, V90I, M184V	0.9%	0
26.2 ^e^	4513	B	L10I, L33F, M46L, I54V, A71I/T, V82A, L90M	M41L, E44D, A62V, D67N, L74V, L100I, K103N, H208Y, L210W, T215Y, H221Y	1.1%	1
26.3	18,213	C	K20R, M36I	A62V, K65R, D67N, V75A/I/T, K101Q, K103N, V106M, E138A, M184V	2.1%	1
26.4	6506	D	M36I	None	1.1%	2
26.5	3656	B	none	V90I, K103N	3.8%	0

^a^ RNA copies/mL. ^b^ determined based on protease (PR)- reverse transcriptase (RT) sequence and Stanford HIVdb. ^c^ Drug resistance-associated mutation (DRM) sites were defined as any position with a potential impact on the penalty score in the Stanford HIVdb algorithm (version 8.5). ^d^ percentage of nucleotides in the VQA Sanger consensus sequence that are mixed, including positions where consensus was not reached. ^e^ same donor virus as 24.5.

## Appendix A

**Table A1:** Assay details.

Laboratory ID	RNA Extraction Method (Specimen Volume)	RT-PCR Amplification Strategy	Negative Control	% of Extracted RNA Used	Coverage (PR, RT aa)	Minimum Read Depth ^a^	Minimum Variant Count ^b^	Analysis Pipeline
1	QIAamp Viral RNA Mini kit (0.14 mL)	RT with primerID, then nested PCR	Water	50%	PR 1–99, RT 34–122 and 152–236	Varies	NA ^c^	TCS pipeline in house
2	ViroSeq RNA extraction kit (0.5 mL)	RT then nested PCR	Water	10%	PR 1–99, RT 1–440	1000	1000	CLC Genomics Workbench and In-house
3	QIAamp Viral RNA Mini kit (1 mL)	One-step RT-PCR then nested PCR	Water	10%	PR 6–99, RT 1–251	1000	50	HyDRA [35]
4	MagnaPure LC (0.5 mL)	One-step RT-PCR, then nested PCR	Water	30%	PR 1–99, RT 1–250	330	NA ^c^	Geneious
5	QIAamp Viral RNA Mini kit (0.14 mL)	One-step RT-PCR then nested PCR	Water	10%	PR 1–99 RT 1–300	100	5	Trim Galore!, HydDRA [35]
6	NucliSENS easyMAG (0.4 mL)	One-step RT-PCR, then nested PCR	Water	~9%	PR 1–99, RT 1–250	100	5	HyDRA [35]
7	QIAamp UltraSens Virus kit (0.5 mL)	Primary RT-PCR, then nested PCR	Fetal bovine serum	16.7%	PR 5–99, RT 1–320	1000	NA ^c^	In-house [36]
8	QIAamp Viral RNA Mini kit (0.14 mL)	One-step RT-PCR then nested PCR	Water	25 %	PR 1–99, RT 1–440	1000	10	PASeq.org [35]
9	EZ1 Advance XL (variable) ^d^	RT then nested PCR	Water	16.7%	PR 1–99, RT 1–240	1000	10 ^e^	Hivmmer [9]
10	NucliSENS easyMAG (0.5 mL)	RT then nested PCR	Water	6.7%	PR 1–99, RT 1–400 or 1–240	100	NA ^c^	MiCall [35]

^a^ minimum number of reads required for data quality assurance. ^b^ minimum number of individual variants required for reporting. ^c^ in some analysis pipelines, the minimum variant count is not specified, although in practice is defined by the minimum coverage and variant proportion. ^d^ volume adjusted based on viral load to contain at least 5000 copies. ^e^ and ≥1% of total coverage at site.

**Table A2:** Nucleotide sequence identity vs. VQA Sanger consensus at different variant thresholds.

	5%	10%	15%	20%
Number	94	94	94	85
Minimum	95.0	95.7	98.2	98.3
Median	98.9	99.6	99.7	99.9
Mean	98.7	99.4	99.6	99.7
Std. Deviation	0.95	0.63	0.41	0.40
Lower 95% CI of mean	98.5	99.2	99.5	99.6
Upper 95% CI of mean	98.9	99.5	99.7	99.8

**Table A3:** Random Effects model testing differences between thresholds.

Comparison	N	Rand Eff Model Mean (SEM)	Rand Eff Model p-value Test = 0	Mean Diff	Median Diff	SD Diff	Min Diff	Max Diff	Paired t *p* value	Sign Test *p* value	Sign Rank *p* value
10% vs 5%	94	0.0062 (0.0016)	0.004	0.0065	0.0044	0.0071	−0.0056	0.0359	0	0	0
15% vs 5%	94	0.0087 (0.0020)	0.002	0.009	0.0056	0.0094	−0.0052	0.0466	0	0	0
15% vs 10%	94	0.0025 (0.00076)	0.01	0.0025	0.0011	0.0048	−0.0028	0.04	0.0000022	0	0
20% vs 5%	85	0.0094 (0.0022)	0.003	0.0097	0.0055	0.01	−0.0034	0.0477	0	0	0
20% vs 10%	85	0.0033 (0.00091)	0.007	0.0034	0.0022	0.0055	−0.0063	0.0411	0.0000002	0	0
20% vs 15%	85	0.00084 (0.0002)	0.003	0.0008	0	0.0017	−0.0075	0.0056	0.0000265	0	0.0000012
